# Molecular cytogenetic identification of small supernumerary marker chromosomes using chromosome microarray analysis

**DOI:** 10.1186/s13039-019-0425-5

**Published:** 2019-03-11

**Authors:** Huili Xue, Hailong Huang, Yan Wang, Gang An, Min Zhang, Liangpu Xu, Yuan Lin

**Affiliations:** Fujian Provincial Key Laboratory for Prenatal diagnosis and Birth Defect, Fujian Provincial Maternity and Children’s Hospital, affiliated hospital of Fujian Medical University, Fuzhou, 350001 Fujian China

**Keywords:** Small supernumerary marker chromosome, Prenatal diagnosis, Fluorescence in situ hybridization, Chromosome microarray analysis, Copy number variation

## Abstract

**Background:**

This study aimed to evaluate the feasibility of chromosomal microarray analysis (CMA) in detecting the origin and structure of small supernumerary marker chromosomes (sSMCs) in prenatal and postnatal cases and to clarify sSMC-related genotype-phenotype correlations.

**Results:**

Thirty-three cases carrying sSMCs were identified by banding cytogenetics. Of these cases, twenty-nine were first characterized by CMA and only two by FISH. The remaining two cases were excluded for their refusal to accept further examination. The chromosomal origins of twenty-two cases were successfully identified, in which pathogenetic copy number variations (PCNVs) were found in sixteen cases, four cases showed variants of uncertain significance (VOUS), one case showed benign CNVs, and one case showed probable PCNVs. For the nine cases with negative CMA results, only one of them contained centromere heterochromatin likely due to its normal phenotype, whereas reasons for the remaining eight cases were uncertain. We also found that CMA results indicating pathogenic abnormalities further affect the rate of pregnancy termination.

**Conclusions:**

This study showed that CMA combined with cytogenetic analysis is particularly effective in identifying sSMCs. However, in order to establish sSMC-related genotype-phenotype correlations, the inclusion of more sSMC cases will be necessary in future studies.

## Background

sSMCs are structurally abnormal chromosomes that cannot be unambiguously identified by G-banding [1]. They are detected in 0.08% of unselected prenatal cases and in 0.20% of prenatal cases presenting fetal abnormalities during ultrasonography [2].

The phenotypes resulting from sSMCs vary widely depending on their origin and size and the effects of sSMCs on phenotype remain unclear. The application of molecular cytogenetic techniques is therefore warranted to identify the origin and structure of sSMCs. Liehr and Weise [2] found that a prenatally-characterized sSMC derived from a non-acrocentric chromosome carried a 30% risk for phenotypic abnormalities.

The resolution of conventional cytogenetic analysis is limited to 5–10 Mb. Furthermore, it is inefficient and costly to use FISH in the identification of chromosomal origin. Recently, CMA has been applied to overcome the limitations of FISH and has been used as a first-tier test in cases involving sSMCs. Despite the fact that the identification of chromosomal origin, size, and degree of mosaicism of sSMCs informs prognosis, prenatal discovery of de novo sSMCs remains a challenge for genetic physicians. Consequently, we analyzed thirty-one cases carrying sSMCs via single nucleotide polymorphism (SNP) arrays and/or FISH in this study, aiming to assess the value of CMA in characterizing sSMCs and to identify the genotype-phenotype correlations associated with these structural abnormalities in chromosomes.

## Methods

### Cases studied

We analyzed a total of thirty-one cases carrying sSMCs via single nucleotide polymorphism (SNP) arrays and/or FISH, aiming to assess the value of CMA in characterizing sSMCs and to identify their genotype-phenotype correlations. From July 2015 to July 2018, thirty-three pre- and post-natal cases were diagnosed as sSMC carriers at the Fujian Provincial Key Laboratory for Prenatal Diagnosis and Birth Defect Research. Two cases refused further investigation; the remaining thirty-one cases, of which seven were from peripheral blood, eight from amniotic fluid, twelve from cord blood, and four from samples containing products of conception, were included in the present study, which was approved by the institutional ethics committee. Informed consent was obtained from all subjects as specified by the Declaration of Helsinki. All patients received pre-test counseling regarding the procedure-related risks and benefits of CMA. Women with positive results were offered comprehensive prenatal counseling by obstetricians and fetal medicine physicians. All prenatal samples obtained by amniocentesis or cordocentesis were analyzed by CMA/FISH and G-banding. In this study, all experiments, i.e., banding cytogenetics, CMA, and FISH, were performed in accordance with relevant guidelines and instructions. CMA was the first analysis method performed on 29 cases to characterize sSMCs and FISH was the first applied in the other two cases (P17, P23).

### Banding cytogenetic analysis

G-banding analysis (C/NOR-banding when necessary) at a resolution of approximately 500 bands was performed on the patients’ peripheral blood according to standard laboratory protocols and ISCN 2016. Amniotic fluid or fetal cord blood samples were obtained according to the invasive procedure protocol [3].

### Chromosome microarray analysis

SNP arrays constitute one type of chromosome microarray analysis (CMA) technology capable of detecting genome-wide CNVs. In 2010, the American College of Medical Genetics issued practice guidelines for CMA, which was recommended as a first-tier test for postnatal patients with multiple congenital anomalies, intellectual disabilities/developmental delays, and autism spectrum disorders [4].

In the present study, genome-wide high resolution CMA was initially performed in twenty-nine cases carrying sSMCs. SNP array analysis was performed using an Affymetrix array (CytoScan® 750 K; Affymetrix/Thermo Fisher Scientific, Santa Clara, CA, USA); the results were analyzed by CHAS software (Affymetrix/Thermo Fisher Scientific), using annotations of the genome version GRCH37. The reporting threshold was set at gains or losses ≥400 kb and loss of heterozygosity (LOH) ≥ 10 Mb [5]. For interpretation of these results, our local database and the following public database were used: DGV (http://projects.tcag.ca/variation/), Cytogenomics Array Group CNV Database (https://www.cagdb.org), Database of Chromosomal Imbalance and Phenotype in Humans using Ensembl Resources database (DECIPHER, http://decipher.sanger.ac.uk/), Online Mendelian Inheritance in Man (OMIM, http://www.omim.org), the CNVs were classified as benign, pathogenic, or VOUS according to the ACMG guidelines [6]. Partial CNVs were further validated by FISH. Parental analysis was performed to interpret VOUS.

### Fish

Prior to July 2015, FISH was performed on six cases. The probes were selected based on the gain region detected by CMA. The majority of commercial probes included chromosome 13/21, 14/22, 15/16, 18, and X centromere probes, the DYZ3 probe located at Yq11.2, and RP11-958H20 probe located at 22q11.1-22q11.2. Interphase/metaphase FISH analysis was performed on cultured lymphocytes and/or amniocytes according to the manufacturer’s protocol.

## Results

### Cytogenetic analysis

Between July 2015 and July 2018, thirty-three pre- and post-natal cases were initially diagnosed as sSMC carriers via banding cytogenetics at Fujian Provincial Key Laboratory for Prenatal Diagnosis and Birth Defect Research. G-banding karyotype analysis showed mosaic marker chromosomes in 20 out of 31 cases (Table 1). Two cases refused further testing. The remaining thirty-one cases gave informed consent to participate in the study; eight samples were obtained by amniocentesis, twelve samples were obtained by cordocentesis, four samples were obtained from products of conception, and seven samples were obtained by collecting peripheral blood.

**Table 1:** Summary of cytogenetic, CMA and FISH findings in sSMCs

Case#	Cytogenetic Results/Mosaicism	De novo/Inherited	CMA results /CNV classification	FISH Results	Clinical features/Reason of study/Chromosome abnormality syndrome	Outcome/Pregnancy outcome
P1	47,XX,+mar The sSMC was C-banding positive, N-banding positive	dn	Fail to detect the chromosome origin	N.D.	AMA, reproductive history of child with autism, fetal BPD and HC values were 2 standard deviations below the mean	TP
P2	mos 47,XY,+mar[5]/46,XY [45]	dn	Arr [GRCh37]7q11.23(74,175,031_74,566,129) × 1,10q11.22q11.23(49,730,919_50,395,827) × 3,10q26.13q26.3(124,383,733_135,426,386) × 2~3,14q23.2(63,970,519_64,284,284) × 1,Yp11.2(7643,38_8,808,561) × 2 VOUS	N.D.	Phenotypically normal couple with missed abortion, the pregnant women had a karyotype of 46,XX,inv.(7) (q22q31.3), CVS revealed a karyotype of mos 47,XY,+mar [5]/46,XY[45]	IA
P3	mos 47,XY,+mar[39]/46,XY[11]	dn	CMA analysis revealed a 16.9 Mb heterozygous duplication in the 8p12-8q11.21 region, encompassing multiple OMIM genes such as *TT12* and *PRKDC* associated with mental delay; *WHSC1L1* is associated with acute myeloid leukemia. According to the ISCA database, this duplicated region can lead to speech loss. PCNV	N.D.	Abnormal second-trimester MSS with a down syndrome risk of 1/120, CVS revealed a karyotype of mos 47,XX,+mar[22]/46,XX[19]	TP
P4	mos 46,X,+mar[33]/45,X [21]/46,X,del(X)(q23)[9]	dn	Arr [GRCh37]2q32.1q32.2(189,194,304_190,487,242) × 3,Xp22.12p11.21(21,782,384_56,905,943) × 1,Xq12q28(65,783,010_155,160,723) × 1 PCNV	N.D.	Phenotypically normal couple with missed abortion twice, CVS revealed a karyotype of mos 46,X,+mar[33]/45,X [21]/46,X,del(X)(q23)[9] TS	IA
P5	47,XX,+mar	N.D.	Fail to detect the chromosome origin	N.D.	Female infertility, cytogenetic analysis of the peripheral blood lymphocytes revealed a karyotype of 47,XX,+mar	FI
P6	mos 46,X,+mar[43]/45,X [7] The sSMC was C-banding negative.	dn	Arr [GRCh37]Xp22.33 or Yp11.32p11.31(168,551_2,693,467 or 118,551_2,643,467) × 4,Xq28 or Yq12(154,941,868_155,233,098) or (59,044,874_59,336,104) × 1,Yp11.31q11.221(2,650,424_18,016,216) × 4,Yq11.221q11.23(18,047,379_28,799,654) × 0 PCNV	N.D.	AMA, amniocentesis revealed a karyotype of mos 46,X,+mar [16]/45,X[30], percutaneous umbilical blood sampling revealed a karyotype of mos 46,X,+mar[43]/45,X [7], detection of SRY and AZF microdeletion showed that SRY positive, AZFa existed, while AZFb and AZFc micro-deletions occurred in the fetal cord blood, ultrasound scan shows it is a male fetus	TP
P7	mos 45,X [24]/46,X,+mar [12]	dn	Fail to detect the chromosome origin	N.D.	M Missed abortion, CVS revealed a karyotype of mos 45,X [24]/46,X,+mar [12] TS	IA
P8	47,XX,+mar	dn	Arr [GRCh37]22q11.1q11.21(16,888,899_18,649,190) × 4 PCNV	47,XX,+mar. ish idic(22)(q11.2)(RP11-958H20++)	AMA, fetal ventricular septal defect, dysplasia of aorta, echogenic intracardic focus, single umbilical artery, amniocentesis revealed a karyotype of 47,XX,+mar. Cat Eye Syndrome	TP
P9	mos 47,XY,+mar [8]/46,XY[42]	dn	Arr [GRCh37]1q21.3(151,917,498_152,861,866) × 3,1q21.3(153,286,503_153,976,253) × 3 VOUS	N.D.	Fetal right subclavicular artery vagus, amniocentesis revealed a karyotype of mos 47,XY,+mar [9]/46,XY[41], percutaneous umbilical blood sampling revealed a karyotype of mos 47,XY,+mar [8]/46,XY[42]	TP
P10	mos 47,XX,+mar [25]/46,XX [25]	dn	Arr [GRCh37]12p11.21q12(31,269,113_42,349,971) × 3 VOUS (likely PCNV)	N.D.	AMA, amniocentesis revealed a karyotype of mos 47,XX,+mar[36]/46,XX [13], percutaneous umbilical blood sampling revealed a karyotype of mos 47,XX,+mar [25]/46,XX [25]	TP
P11	mos 45,X [24]/46,X,+mar [26]	dn	Arr [GRCh37]Xp22.33q11.1(168,551_62,006,469) × 1,Xq21.31q28(87,685,781_155,233,098) × 1 PCNV	mos 45,X [24]/46,X,+mar [26].ish 45,X (DXZ1 × 1,SRY × 0[6]46,X,+mar.ish r(X)(DXZ1+) [8]	amniocentesis revealed a karyotype of mos 46,X,+mar[32]/45,X [28], percutaneous umbilical blood sampling revealed a karyotype of mos 45,X [24]/46,X,+mar [26] TS	TP
P12	mos 46,X,+mar[36]/46,XX [14]	dn	Arr [GRCh37]Xp22.33p11.21(168,551_56,661,860) × 1,Xq21.1q28(79,764,187_155,233,098) × 1 PCNV	N.D.	AMA, thickened nuchal fold (NF),strong echo in left ventricle, single umbilical artery, amniocentesis revealed a karyotype of mos 46,X,+mar[36]/46,XX [14] TS	TP
P13	mos 46,X,+mar [23]/46,XY [27]	dn	Arr [GRCh37]Xp22.33 or Yp11.32(168,551_2,019,878 or 118,551_1,969,878) × 3,Yp11.31q11.221(2,650,424_16,094,327) × 2,Yq11.221q11.23(16,189,079_28,799,654) × 0 PCNV	N.D.	Amniocentesis revealed a karyotype of mos 46,X,+mar[55]/46,XY[45], percutaneous umbilical blood sampling revealed a karyotype of mos 46,X,+mar [23]/46,XY [27] Fetal level III ultrasound findings were unremarkable	TP
P14	mos 47,XY,+mar [17]/46,XY[34]	dn	Arr[GRCh37]12p13.33p11.1(173,786_34,759,042) × 2~3,20p13p11.1(186,793_26,129,447) × 2~3 PCNV	N.D.	AMA, amniocentesis revealed a karyotype of mos 47,XY,+mar [13]/46,XY[37], percutaneous umbilical blood sampling revealed a karyotype of mos 47,XY,+mar [17]/46,XY[34] Mitral regurgitation + ~ + on fetal ultrasound	TP
P15	47,XX,+mar	dn	Arr[GRCh37]12p13.33p11.1(173,786_34,835,641) × 4 PCNV	N.D.	AMA, enlarged nuchal translucency on fetal ultrasound Pallister-Killian syndrome	TP
P16	mos 45,X[43]/46,X,+mar [7]	dn	Arr[GRCh37]Yq11.221q11.222(17,082,004_19,927,040) × 2,Yq11.222q11.23(21,035,823_28,799,654) × 0 PCNV	mos 45,X[43]/46,X,+mar [7].ish 45,X(DXZ1x1,DYZ3x0) [22]/46,X,idic(Y)(q11.2?)(DXZ1x1,DYZ3× 2) [2]/ 47,X,idic(Y)(q11.2?)× 2(DXZ1x1,DYZ3x4) [1]	Abnormal second-trimester MSS for down syndrome with a risk of 1/238, fetal tricuspid regurgitation, wide right pulmonary artery diameter on fetal ultrasound, amniocentesis revealed a karyotype of mos 45,X[43]/46,X,+mar [7] TS	TP
P17	47,XY,+mar	dn	N.D.	47,XX,+mar. ish r(18)(D18Z1+) VOUS	Her daughter (P23) had a karyotype of mos 47,XX,+mar[44]/46,XX [6] Phenotypically normal	H
P18	47,XX,+mar	dn	Arr[GRCh37]16p11.2(32,024,388_33,800,323) × 3 Benign (refused trios analysis)	N.D.	AMA, amniocentesis revealed a karyotype of 47,XX,+mar	CP
P19	mos 45,X[33]/46,X,+mar [18]	dn	Arr[GRCh37](X) × 1 PCNV	N.D.	Enlarged nuchal translucency on fetal ultrasound, NIPT suggested sex chromosome aneuploidy, amniocentesis revealed a karyotype of mos 45,X[33]/46,X,+mar [18] TS	TP
P20	mos 47,XY,+mar [2]/46,XY[48]	dn	Arr[GRCh37]12p13.33p11.1(173,786_34,835,641) × 3 PCNV	N.D.	Amniocentesis revealed a karyotype of mos 47,XY,+mar [5]/46,XY[45], percutaneous umbilical blood sampling revealed a karyotype of mos 47,XY,+mar [2]/46,XY[48] Trisomy 12p13.33p11.1	TP
P21	46,XX,-18,+mar	dn	Arr[GRCh37]18p11.32p11.31(136,227_3,348,254) × 1, 18p11.31p11.21(3,350,736_13,083,388) × 3,18p11.21(13,090,666_15,170,636) × 1,18p11.21q21.31(15,181, 207_54,008,143) × 3,18q21.31q23(54,020,488_78,013,728) × 1 PCNV	N.D.	Fetal aorta constriction, pulmonary artery stenosis after dilation, ventricular septal defect on fetal ultrasound, NIPT suggests partial deletion in chromosome 18	TP
P22	47,XX,+mar	dn	Fail to detect the chromosome origin	N.D.	Female infertility for 4 years	PI
P23	mos 47,XX,+mar[44]/46,XX [6]	MI	N.D.	mos 47,XX,+mar[44]/46,XX [6].ish mar(D18Z1+) [14]/46,XX [1] VOUS	Arcuate uterus, Mternal karyotype was 47,XX,+mar, presented with an adverse pregnancy outcome (premature labor occurred at 28 weeks of gestation, and a 1015 g female baby with hydrocephalus and severe asphyxia was delivered stillborn), amniocentesis revealed a karyotype of mos 47,XX,+mar[44]/46,XX [6]	SI
P24	mos 45,X[30]/46,X,+mar [16]	dn	Arr[GRCh37]19p13.3(633,754_1,230,420) × 3,Xp22.33p11.21(168,551_57,994,702) × 1,Xp11.21q12(58,053,772_66,837,037) × 1,Xq12q28(66,896,948_155,233,098) × 1 PCNV	N.D.	PA, normal stature, infantile uterus, seizures, abnormal thyroid function TS	PA
P25	mos 47,XX,+mar [14]/46,XX [36]	dn	Fail to detect the chromosome origin	mos 47,XX,+mar [14]/46,XX[36].ish mar(D15Z1-)[15]46,XX [15]	Abnormal second-trimester MSS for down syndrome with a risk of 1/200,amniocentesis revealed a karyotype of mos 47,XX,+mar [15]/46,XX[35], percutaneous umbilical blood sampling revealed a karyotype of mos 47,XX,+mar [14]/46,XX[36]	CP
P26	mos 45,X[45]/46,X,+mar [5]	dn	Arr[GRCh37](X) × 1 PCNV	N.D.	PA, orally -administered climen can still menstruate at 20 years old but small amount, short stature (147 cm), bilateral breast development is good, infantile uterus TS	PI
P27	mos 47,XX,+mar[39] /46,XX [11]	dn	Arr[GRCh37]9p24.3q13(208,454_68,216,577) × 4 PCNV	N.D.	FGR at 28^+ 3^ weeks of gestation, percutaneous umbilical blood sampling revealed a karyotype of mos 47,XX,+mar[39]/46,XX [11] Mosaic tetrasomy 9p	TP
P28	47,XY,+mar	dn	Fail to detect the chromosome origin	N.D.	His wife underwent RSA, cytogenetic analysis of the peripheral blood lymphocytes revealed a karyotype of 47,XY,+mar	SS
P29	47,XN,+mar	dn	Fail to detect the chromosome origin	N.D.	AMA, posterior fossa cistern widened, renal pelvis separated on fetal ultrasound, amniocentesis revealed a karyotype of 47,XN,+mar	CP
P30	47,XN,+mar The sSMC was C-banding positive, N-banding positive.	dn	Fail to detect the chromosome origin	N.D.	Abnormal second-trimester MSS for down syndrome with a down syndrome risk of 1//263, amniocentesis revealed a karyotype of 47,XN,+mar. Fetal level III ultrasound findings were unremarkable	CP
P31	mos 47,XY,+mar [3]/46,XY[58]	dn	Fail to detect the chromosome origin	N.D.	AMA, poliomyelitis, fetal HL and FL values were less than gestational weeks on fetal ultrasound at 22 weeks of gestation, amniocentesis revealed a karyotype of mos 47,XY,+mar [3]/46,XY[58]	CP

*Dn* de novo, *N. D* not done, *AMA* advanced maternal age, *FGR* fetal growth restriction, *TP* termination of pregnancy, *sSMC* small supernumerary marker chromosome, *PCNV* pathogenic copy number variation, *CP* continue the pregnancy, *TS* turner syndrome, *MSS* maternal serum screening, *NIPT* non-invasive prenatal testing, *CVS* chorionic villus sampling, *IA* induced abortion, *SS* secondary sterility, *PI* primary infertility, *PA* primary infertility, *SI* secondary infertility, *H* healthy, *FI* female infertility, *MI* maternally inherited, *RSA* recurrent spontaneous abortion, *BPD* biparietal diameter, *HC* head circumference, *HL* humerus length, *FL* femur length, *AZF* azoospermia factor, *SRY* sex-determining region on the Y chromosome

### CMA and FISH analysis

In this study, thirty-three cases bearing sSMCs were initially identified via banding cytogenetics; however, due to the refusal of two cases for further study, twenty-nine cases first underwent CMA and only two underwent FISH. The chromosomal origins of twenty-two cases were successfully identified, of which sixteen were pathogenetic copy number variations (PCNVs), four were variations of uncertain clinical significance (VOUS), and two were benign CNVs. For the nine cases with negative CMA results, only one contained centromere heterochromatin—most likely due to its normal phenotype—whereas the reasons for the remaining eight cases were uncertain. We also found that CMA results indicating pathogenic abnormalities affected the rate of pregnancy termination. The clinical characteristics and pregnancy outcomes of thirty-one sSMC carriers are summarized in Table 1.

Of twenty cases with positive CMA results, three isochromosome sSMCs originated from chromosomes 22 [P8] and Y (P15 and P16), respectively. Two ring marker chromosomes were derived from chromosomes X (P11) and 18 (P17), respectively. Four originated from chromosome 12p (P10, P14, P15, and P20), of which three were derived from 12p13.33p11.1 (all except for P10). One case originated from chromosome 22 containing the cat eye syndrome (CES) critical region (22q11.1q11.21) (P8). Seven cases originated from chromosomes 10 (P2), 8 (P3), 1 (P9), 2 (P4), 16 (P18), 18 (P21), and 19 (P24), respectively. Eight cases carried complex sSMCs (P2, P4, P6, P11, P12, P13, P14, and P24); case P2 had a rare recombination of 7q11.23, 10q11.22q11.23, 10q26.13q26.3, 14q23.2, and Yp11.2 that had not been reported previously. In case P4, CMA analysis revealed one gain and two losses in different chromosomes, in addition to a 1.3 Mb gain at 2q32.1q32.2 that involved five OMIM genes [*GULP1(*608165)*, DIRC1*(606423)*, COL3A1*(120180)*, COL5A2*(120190), and *SLC40A11*(604653)] and an additional 125 Mb loss at Xp22.12-qter that involved four OMIM genes (*MECP2*, *GRIA3*, *AFF2*, and *MAMLD1*). In addition to cat eye syndrome (P8) [7], we also detected CNVs associated with tetrasomy 9p (P27) [8] and Pallister-Killian syndrome (P15) [9], as well as Turner syndrome (TS) [10].

In the nine cases with negative CMA results, subsequent FISH analysis of P25 utilizing probe D15Z1/D16Z3 revealed no signal in metaphase cells, while further investigation via FISH was not performed on the remaining eight cases due to limited quantity of specimen or patient refusal.

### Inheritance analysis and pregnancy outcomes

Parental karyotype analysis showed that in one case, the abnormality was inherited from her unaffected mother and in the remaining thirty cases, the CNVs occurred de novo. In the present study, the five couples (P18, P25, and P29–P31) receiving fetal prognoses that were more likely to be favorable decided to continue their pregnancies. At present, these pregnancies are ongoing. The parents of the other 15 PCNV cases (P3, P6, P8–P16, P19–21, and P27) opted to terminate their pregnancies.

## Discussion

In our study, karyotype analysis identified thirty-three cases carrying sSMCs of which thirty-one cases were further identified by CMA and/or FISH, including twenty-one cases in prenatal diagnosis; chromosomal origin and gene content were successfully identified in a total of twenty-two cases (Table 1).

Aside from the detection of CNVs, uniparental disomy, and chimeric chromosomes, CMA rapidly provides accurate information on the origin and structure of sSMCs in a single assay and is especially effective for complicated prenatal cases. In our study, twenty-one cases who carried sSMCs based on prenatal diagnosis were identified by SNP-array and/or FISH analyses; chromosomal origin was successfully identified in sixteen of these cases, thus circumventing uncertainty and blind termination of pregnancy. In case P27, CMA analysis revealed a four-copy gain of 68 Mb in 9p24.3q13 and cytogenetic analysis of the fetal cord blood revealed a karyotype of mos 47,XX,+mar[39]/46,XX[11], consistent with 78% mosaicism; hence, the fetus of case P27 was diagnosed with mosaic tetrasomy 9p [8]. Besides tetrasomy 9p, we also identified CNVs associated with cat eye syndrome [7] and Pallister-Killian syndrome [9], as well as TS [10]. Patients with these syndromes display a range of physical and mental disabilities, including developmental delays.

CMA can identify the origin and size of sSMCs in one test and is especially useful for complex sSMCs. In our study, eleven cases carried complex sSMCs based on CMA analysis. In case P2, banding cytogenetics of the peripheral blood lymphocytes showed a karyotype of mos 47,XY,+mar[5]/46,XY[45]. CMA analysis revealed mosaic sSMCs derived from 10q11.22q11.23 (0.6 Mb), 10q26.13q26.3 (11 Mb), and Yp11.2 (1.1 Mb), respectively. To the best of our knowledge, case P2 carrying a complex sSMC involving chromosomes 7, 10, 14, and Y, respectively, had not been reported previously. However, subsequent confirmatory FISH was not performed due to refusal of further analysis by the pregnant woman. In case P14, in addition to a 34.5 Mb gain in the 12p13.33p11.1 region involving 227 OMIM genes and 50–60% mosaicism for genomic imbalance, CMA analysis revealed another 25.9 Mb gain in the 20p13p11.1 region involving 126 OMIM genes and 50–60% mosaicism for genomic imbalance. In case P21, CMA analysis revealed three losses and two gains in chromosome 18, including a 3.2 Mb loss at 18p11.32p11.31, a 2.0 Mb loss at 18p11.21, and a 23.9 Mb loss at 18q21.31q23 and a 9.7 Mb gain at 18p11.31p11.21 and a 38.8 Mb gain at 18p11.21q21.31, respectively, according to the DECIPHER database and the reports of other studies [11–15]. These fragments are associated with fetal abortion, intrauterine stillbirth or multiple malformations, developmental delay, mental delay, and other abnormalities in neonates; thus, the parents opted to terminate their pregnancies after comprehensive genetic counseling. The cases described above with abnormal clinical phenotypes suggest that these sSMCs are likely to be PCNVs; therefore, these sSMCs may be responsible for the phenotypes observed in these cases.

**Fig. 1:**
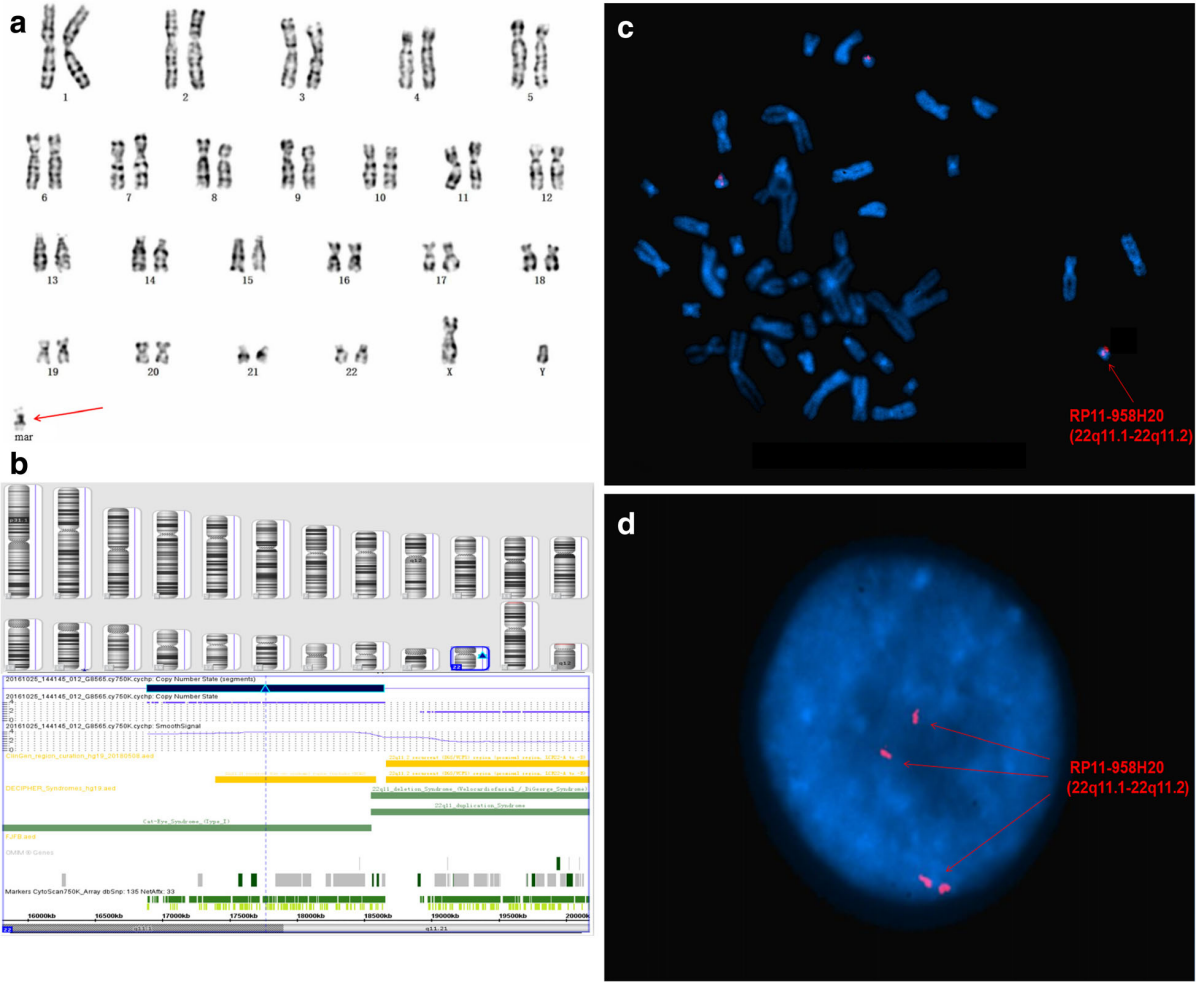
Cytogenetic and molecular results of case P8. The marker is highlighted by a red arrow in **a**, **c** and **d**. **a** In all of the studied cells, G-banding revealed a karyotype 47,XX,+mar. **b** The sSMC of case P8 characterized after SNP array covering 1.7 Mb [Arr[GRCh37]22q11.1q11.21(16,888,899_18,649,190) × 4] in chromosome 22. **c** and **d** Confirmatory metaphase and interphase FISH results of this sSMC using the RP11-958H20 probe specific for 22q11.1-22q11.2 revealed that all the cells had four distinct signals

**Fig. 2:**
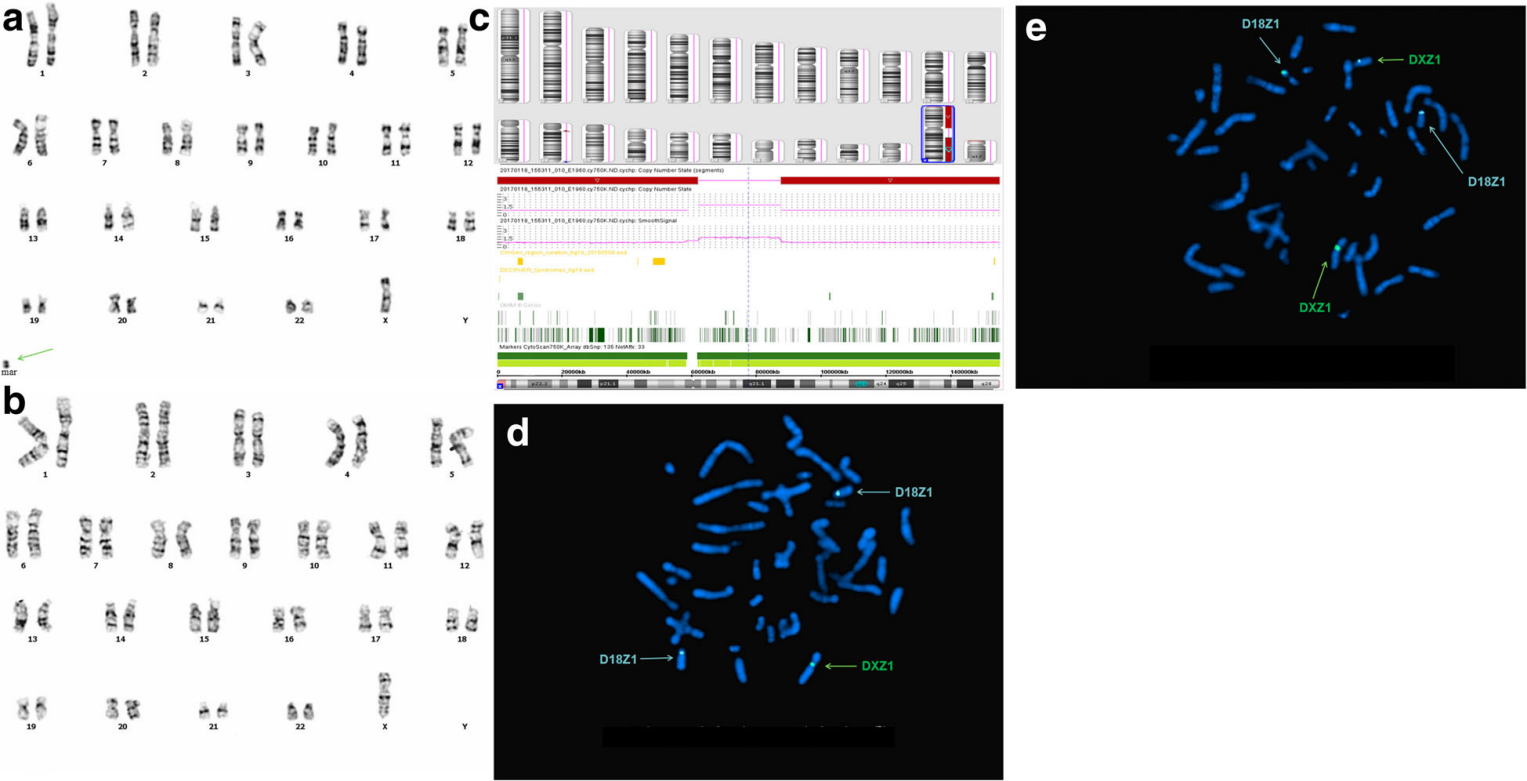
Cytogenetic and molecular results of case P11. The marker is highlighted by a green arrow in a, d and e. **a** In 26 out of the 50 studied cells, G-banding revealed a karyotype 46,X,+mar. **b** In the other 24 cells, G banding revealed a karyotype 45,X. **c** The sSMC of case P11 characterized after SNP array covering 62 Mb [arr[GRCh37]Xp22.33q11.1(168,551_62,006,469) × 1] in chromosome X and 68 Mb [arr[GRCh37]Xq21.31q28(87,685,781_155,233,098) × 1] in chromosome X. **d** and **e** FISH using DXZ1 and D18Z1 probes for X and 18 chromosomes centromeres (green and turquoise signals, respectively). Confirmatory metaphase FISH results of this sSMC using the DXZ1 probe specific for X centromeric probe (CEP) revealed that 57% of the cells had two distinct signals

**Fig. 3:**
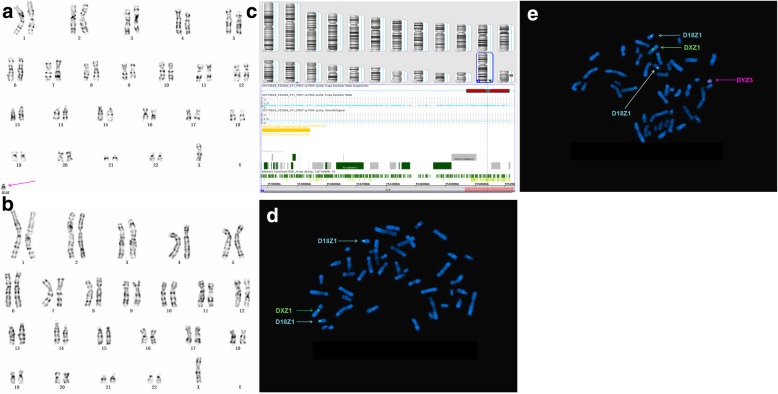
Cytogenetic and molecular results of case P16. The marker is highlighted by a pink arrow in a and e. **a** In thirteen out of the one hundred studied cells, G-banding revealed a karyotype 46, X,+mar. **b** In the other eighty-seven cells, G banding revealed a karyotype 45, X. **c** The sSMC of case P16 characterized after SNP array covering 2.8 Mb [arr [GRCh37]Yq11.22q11.222(17,082,004_19,927,040) × 2] in chromosome Y and 7.7 Mb [arr[GRCh37]Yq11.222q11.23(21,035,823_28,799,654) × 0] in chromosome Y. **d** and **e** FISH using DXZ1 and DYZ3 probes for X and Y chromosomes centromeres (green and red signals, respectively). Confirmatory metaphase FISH results of this sSMC using the DYZ3 probe specific for Yq11.2 revealed that 4% of the cells had four distinct signals and 8% of the cells had two distinct signals

CMA can also detect additional underlying CNVs missed by banding cytogenetics in a single assay. P24 manifested with primary amenorrhea, immature uterus, epilepsy, and abnormal thyroid function; her stature was normal (158 cm), her karyotype was mos 45,X[30]/46,X,+mar[16], and she was diagnosed with TS. Subsequently, CMA detected an additional gain of ~ 597 Kb in 19p13.3, encompassing 20 OMIM genes including *KISS1R* and *STK11* which are associated with lung oncogenesis or remote metastasis resulting from the deletion of 19p13.3 [16]. Although there is no reported correlation between 19p13.3 duplication and TS, this fragment has been confirmed as a PCNV. This finding suggested that certain patients with sSMCs may carry additional chromosomal abnormalities that may have been missed if CMA was not performed. According to a previous study [17], CMA is especially effective in characterizing SMCs due to its high accuracy and resolution, as well as in detecting and identifying CNVs; though CMA can provide more detailed and useful information in establishing sSMC-related phenotype-genotype correlations and assessing accurate prognoses during comprehensive genetic counseling, it is unable to fully replace other techniques including cytogenetic analysis and FISH. In our study, CMA and FISH validated each other; indeed, CMA cannot be accurately interpreted without the results of banding cytogenetics and FISH. In cases P8 (Fig. 1), P11 (Fig. 2), and P16 (Fig. 3), all three technologies were required to fully characterize the sSMCs.

Armanet et al. [18] observed that sSMCs can perturb meiosis, involving both a gene dosage increase effect and a mechanical effect; therefore, they postulated that sSMCs are implicated in human infertility. Manvelyan et al. [19] reported 7% of sSMCs in males presented decreased sperm parameters. Olszewska et al. [20] also postulated a position effect of sSMCs in infertile male patients. In the current study, seven non-consanguineous infertile couples (P4, P5, P7, P22, P23, P26, and P28) were referred to a genetic clinic because of primary/secondary infertility. Three couples (P4, P7, and P28) manifested spontaneous abortion or missed abortion, though the results of gynecological and infectious investigations on the couples were normal. In case P4, CMA analysis revealed one gain and two losses on different chromosomes, in addition to a 1.3 Mb gain at 2q32.1q32.2 that involved five OMIM genes [*GULP1(*608165)*, DIRC1*(606423)*, COL3A1*(120180)*, COL5A2*(120190), and *SLC40A11* (604653)] and an additional 125 Mb loss at Xp22.12-qter that involved four OMIM genes (*MECP2*, *GRIA3*, *AFF2*, and *MAMLD1*), which are associated with abnormal embryo development, mental delay, premature ovarian failure and gender dysplasia, as well as embryonic death or spontaneous abortion. Three genes (*COL3A1, COL5A2*, and *SLC40A1*) have also been associated with autosomal dominant disorders (Ehlers-Danlos syndrome classical type, hemochromatosis type 4, and muscle hypertrophy) [21]. However, in four cases (P5, P7, P22, and P28), CMA failed to characterize the origins of the sSMCs. The most likely reasons were that the sSMCs were formed exclusively of heterochromatin and/or low level mosaicism (< 20%) [22]; another possibility is that the Affymetrix CytoScan® 750 K chip may have lacked sufficient probes to map the sSMCs; moreover, the sSMC was not causative for the phenotype, which may have been the result of an undetected factor. These undetected sSMCs were much more likely to have a favorable prognosis due to the lack of euchromatin [23]. However, such sSMCs can still arrest meiosis and eventually induce fertility disorders [24].

sSMCs are present in abnormal karyotypes such as the TS karyotype (sSMC^T^) [10]. In the present study, seven cases with sSMC^T^ were detected; the sSMC^T^ carriers mostly presented with mosaicism and almost all were confirmed to originate on chromosomes X or Y, which were frequently associated with TS phenotypes and are generally characterized by greater uncertainty and worse prognoses [25]. In eight cases, the CNVs were found to have occurred de novo after parental karyotype analysis. The couples of these prenatal cases opted to terminate their pregnancies due to chromosomal abnormalities.

In prenatal diagnosis, VOUS may cause considerable anxiety in couples undergoing investigation. In this study, the rate of detection for VOUS was 1.6% (5/31), similar to that reported in the literature [26]. Of these thirty-one cases carrying sSMCs, the origins of the marker chromosomes were characterized in twenty-two cases, of which 16 were PCNVs, four were VOUS, one was a benign CNV, and one was a likely PCNV. As the widespread use of CMA for genetic disorders increases, the rate of detection for VOUS will decrease substantially.

More than half of sSMC carriers present with mosaicism [27] and < 20% of mosaicism is misdiagnosed by CMA. In addition, if the sSMC in question consists of heterochromatin exclusively, CMA would be unable to detect the origin of the marker chromosome. In this study, CMA failed to characterize nine cases, of which one (P1) presented no remarkable clinical findings. In case P1, CMA failed to detect the origin of the sSMC, which was C-banding positive and N-banding positive; these banding cytogenetics results suggested that the marker may derive from an acrocentric chromosome. However, fetal level III ultrasound examination revealed that fetal biparietal diameter (BPD) and head circumference (HC) values were less than the normal predictive values of 2 standard deviations; given that this couple already had a son with autism, they refused further analysis and opted to terminate the pregnancy. In case P25, the sSMC was identified in the fetus though the parental karyotypes were normal; the most likely reason that the phenotype of the fetus was normal may be that the marker only contained heterochromatin. FISH analysis using probe D15Z1/D16Z3 showed no signal on the sSMC.

The possible mechanisms of sSMC formation include gamete complementation, post-fertilization errors, as well as trisomy and monosomy rescue events [28]. Liehr and Weise [2] showed that approximately 70% of de novo sSMC carriers were clinically normal. In the present study, only one case (P23) was maternally inherited; the other thirty cases were de novo, most of whom were phenotypically abnormal. The main reason for variance in the results might be due to the small sample size. We also found that 64.5% (20/31) were mosaics based on chromosome analysis. Most sSMCs are de novo—less than 30% are inherited [2]. In case P23, a 28-year-old female was referred to the genetic clinic with an arcuate uterus and a history of abnormal pregnancy (premature labor occurred at 28 weeks of gestation and a 1015 g female baby with hydrocephalus and severe asphyxia was delivered stillborn); the result of the maternal karyotype was 47,XX,+mar and sSMC(18) was initially identified in the woman and then detected in her mother (P17) with a normal phenotype. We can thereby conclude that the sSMC was inherited maternally. Metaphase FISH analysis using the satellite probes CEP18, CEPX, and CEPY yielded a result of mos 47,XX,+mar[44]/46,XX [6].ish mar(D18Z1+) [14]/46,XX [1]; therefore, this sSMC originated from chromosome 18. However, her parents refused to allow further trio testing, so the origin and genomic content of the sSMC remained unclear.

In this study, we found that sixteen cases carrying sSMCs resulted in termination of pregnancy, including 15 PCNV cases. PCNVs and chromosome abnormalities, as well as other ultrasound abnormalities revealing worse prognoses, are the main reasons for termination of pregnancy. We also found that the couples were more likely to continue their pregnancies when CMA failed to detect the origin of sSMCs in fetuses. These findings highlight the importance of CMA in identifying sSMCs for improved genetic counseling.

## Conclusions

Thirty-one sSMC cases were characterized by cytogenetic analysis, CMA, and FISH. Ultimately, the chromosomal origins for twenty-two of these cases were successfully identified. In conclusion, CMA combined with cytogenetic analysis is particularly effective in rapidly identifying sSMCs with enhanced sensitivity. These methods are useful for assessing the prognosis of fetuses carrying sSMCs. Further studies involving more cases are necessary in order to establish the sSMC-related genotype-phenotype correlations.

## Abbreviations

AMA: Advanced maternal age
BPD: Biparietal diameter
CMA: Chromosome microarray analysis
CNVs: Copy number variations
CP: Continue the pregnancy
CVS: Chorionic villus sampling
Dn: De novo
FGR: Fetal growth restriction
FISH: Fluorescence in situ hybridization
FL: Femur length
HC: Head circumference
HL: Humerus length
MSS: Maternal serum screening
N.D: Not done
NIPT: Non-invasive prenatal testing
NOR: Nucleolar organizing region
SNP: Single nucleotide polymorphism
sSMC: Small supernumerary marker chromosome
TP: Termination of pregnancy
TS: Turner syndrome
VOUS: Variants of unknown significance
